# Temporal Trends in Cancer Mortality Among People Younger Than 50 Years in Norway

**DOI:** 10.1001/jamanetworkopen.2026.29437

**Published:** 2026-08-14

**Authors:** Luís Nunes, Arild Nesbakken, Ragnhild A. Lothe, Anita Sveen

**Affiliations:** 1 Department of Molecular Oncology, Institute for Cancer Research, Oslo University Hospital, Oslo, Norway; 2 Institute of Clinical Medicine, Faculty of Medicine, University of Oslo, Oslo, Norway; 3 Department of Gastrointestinal Surgery, Oslo University Hospital, Oslo, Norway

## Abstract

This cohort study evaluates trends in cancer mortality among individuals aged 20 to 49 years in Norway from 1996 to 2024.

## Introduction

Colorectal cancer (CRC) has become the leading cause of cancer-related death among individuals younger than 50 years in the United States and is the only major cancer type with increased mortality.^1^ Increasing incidence of early-onset CRC is also observed in other countries, including Norway,^2,3^ but the corresponding mortality within a public health care system is unknown. We analyzed cancer mortality among individuals younger than 50 years in Norway the last 3 decades.

## Methods

For this cohort study, annual numbers of cancer deaths in Norway from 1996 through 2024 were obtained from the Association of the Nordic Cancer Registries (NORDCAN, version 9.5.1-02.2026; accessed April 2026).^4,5^ In this period, the current version of the *International Statistical Classification of Diseases and Related Health Problems, Tenth Revision *(*ICD-10*) was used. Corresponding population data by sex and calendar year were obtained from the Cancer Registry of Norway.^6^ Age-standardized mortality rates per 100 000 person-years were calculated using the Segi 1960 world standard population. Standard errors were computed assuming a Poisson distribution. Temporal patterns were analyzed with the Joinpoint regression program version 6.0.1 (National Cancer Institute) for all cancer deaths, the 5 cancer types with the largest number of deaths, and colon and rectal cancers separately. A maximum of 3 joinpoints were allowed. Model selection was performed with permutation testing. Mean annual percentage changes were estimated for the most recent 10-year period (2015-2024). Statistical significance was assessed using 2-sided tests, with *P* < .05 considered significant. STROBE reporting guidelines were followed. Ethical approval and informed consent were not required based on guidelines from the Regional Committee for Medical and Health Research Ethics because the study involved anonymized public-use data.

## Results

Overall, 13 250 individuals (56.2% female) aged 20 to 49 years died from cancer in Norway between 1996 and 2024, representing 4.2% of all cancer-related deaths in this period. The age-standardized mortality rate decreased by 56.0% in the young patient group, from 30.2 per 100 000 individuals in 1996 to 13.3 in 2024, corresponding to a mean annual decline of 2.8% (95% CI, −3.0% to −2.6%). A corresponding decline in the last 10-year period was observed for each leading cause of cancer death (Figure), including brain cancer (−1.6%; 95% CI, −2.3% to −0.9%) and lung cancer (−5.4%; 95% CI, −6.1% to −4.8%) in both sexes, breast cancer (−3.6%; 95% CI, −4.3% to −3.0%) in female patients, and skin melanoma (−3.1%; 95% CI, −4.8% to −1.7%) and pancreatic cancer (−1.6%; 95% CI, −3.1% to −0.1%) in male patients (Table). The point estimate for cervical cancer in female patients was negative, but the finding was not statistically significant (−2.2%; 95% CI, −7.9% to 2.3%).

**Figure.**
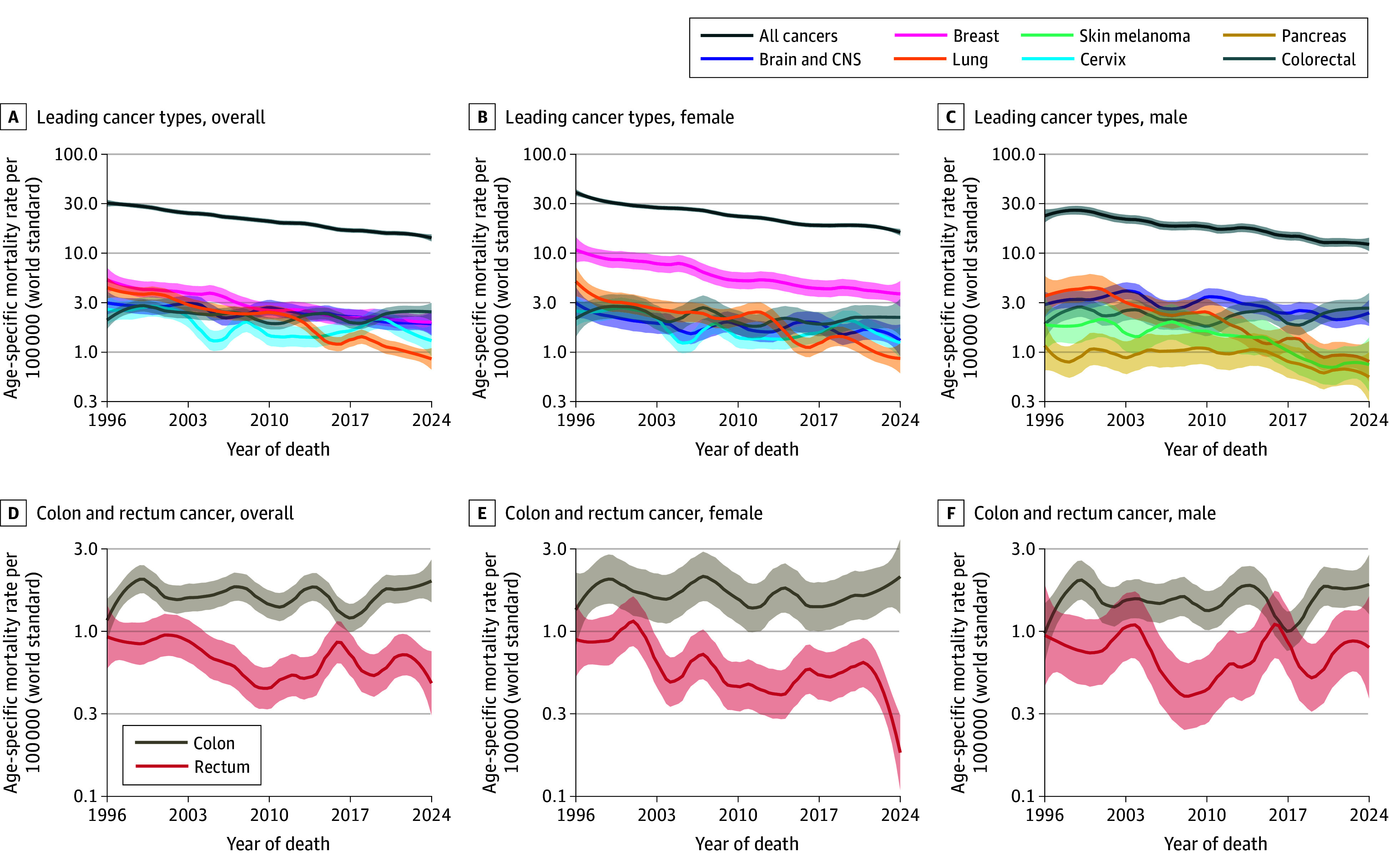
Line Graphs of Mortality Trends for Leading Cancer Types Among Individuals Younger Than 50 Years in Norway, 1996-2024 Dark blue lines include all cancer types except nonmelanoma skin cancer. The bottom panels display colon and rectal cancers separately. Curves were smoothed using locally estimated scatterplot smoothing (span, 0.3), and shading represents 95% CIs. CNS indicates central nervous system.

**Table.** Mortality Trends for Major Cancers and Colorectal Subsites in Individuals Younger Than 50 Years in Norway^a^

Cancer type	Trend segments, No.	Period	APC (95% CI)	APC 2015-2024, mean (95% CI)
All cancers^b^	1	1996-2024	−2.8 (−3.0 to −2.6)^c^	−2.8 (−3.0 to −2.6)^c^
Male	1	1996-2024	−2.8 (−3.2 to −2.4)^c^	−2.8 (−3.2 to −2.4)^c^
Female	1	1996-1999	−7.3 (−9.2 to −3.4)^c^	−2.6 (−2.8 to −2.2)^c^
	2	1999-2024	−2.6 (−2.8 to −2.2)^c^	
Colorectal	1	1996-2024	−0.3 (−1.0 to 0.4)	−0.3 (−1.0 to 0.4)
Male	1	1996-2024	0.1 (−0.8 to 1.0)	0.1 (−0.8 to 1.0)
Female	1	1996-2024	−0.8 (−1.9 to 0.4)	−0.8 (−1.9 to 0.4)
Breast (female)	1	1996-2024	−3.6 (−4.3 to −3.0)^c^	−3.6 (−4.3 to −3.0)^c^
Cervix (female)	1	1996-2014	−4.3 (−8.2 to −0.02)^c^	−2.2 (−7.9 to 2.3)
	2	2014-2020	9.0 (−8.3 to 22.7)	
	3	2020-2024	−14.5 (−31.1 to 0.8)	
Brain and CNS	1	1996-2024	−1.6 (−2.3 to −0.9)^c^	−1.6 (−2.3 to −0.9)^c^
Male	1	1996-2024	−1.6 (−2.5 to −0.7)^c^	−1.6 (−2.5 to −0.7)^c^
Female	1	1996-2024	−1.7 (−2.7 to −0.6)^c^	−1.7 (−2.7 to −0.6)^c^
Lung	1	1996-2024	−5.4 (−6.1 to −4.8)^c^	−5.4 (−6.1 to −4.8)^c^
Male	1	1996-2024	−5.8 (−7.1 to −4.8)^c^	−5.8 (−7.1 to −4.8)^c^
Female	1	1996-2024	−4.9 (−6.0 to −4.1)^c^	−4.9 (−6.0 to −4.1)^c^
Skin melanoma	1	1996-2009	1.7 (−0.6 to 5.9)	−7.3 (−10.8 to −5.3)^c^
	2	2009-2024	−7.3 (−10.9 to −5.3)^c^	
Male	1	1996-2024	−3.1 (−4.8 to −1.7)^c^	−3.1 (−4.8 to −1.7)^c^
Female	1	1996-2009	4.3 (1.2 to 9.6)^c^	−9.0 (−13.5 to −6.5)^c^
	2	2009-2024	−9.0 (−13.5 to −6.5)^c^	
Pancreas	1	1996-2024	−1.2 (−2.5 to 0.1)	−1.2 (−2.5 to 0.1)
Male	1	1996-2024	−1.6 (−3.1 to −0.1)^c^	−1.6 (−3.1 to −0.1)^c^
Female	1	1996-2024	−0.8 (−3.1 to 1.7)	−0.8 (−3.1 to 1.7)
Colon	1	1996-2024	0.1 (−0.9 to 1.3)	0.1 (−0.9 to 1.3)
Male	1	1996-2024	0.3 (−0.9 to 1.5)	0.3 (−0.9 to 1.5)
Female	1	1996-2024	−0.03 (−1.3 to 1.5)	−0.03 (−1.3 to 1.5)
Rectum	1	1996-2024	−1.3 (−2.8 to 0.1)	−1.3 (−2.8 to 0.1)
Male	1	1996-2024	−0.2 (−1.9 to 1.5)	−0.2 (−1.9 to 1.5)
Female	1	1996-2024	−2.8 (−4.4 to −1.3)^c^	−2.8 (−4.4 to −1.3)^c^

Abbreviations: APC, annual percentage change; CNS, central nervous system.

^a^ Models allowed up to 3 joinpoints (4 trend segments); a maximum of 3 segments was identified. Trend segments are row-specific joinpoint-defined periods, and thus calendar years may differ across cancer types and subgroups.

^b^ All cancer types except nonmelanoma skin cancer.

^c^ APC or mean APC (for the most recent 10-year period) in age-standardized mortality rates (Segi 1960 world standard population) is significantly different from zero (*P* < .05).

The only exception was CRC, which has had stable mortality since 1996 (−0.3%; 95% CI, −1.0% to 0.4%). Consequently, CRC has been the leading cause of cancer death in the young patient population since 2020. Notably, there has not been any increase in CRC mortality among young patients, and subgroup analysis by patient sex and tumor site showed a decrease in mortality among female patients with rectal cancer (−2.8%; 95% CI, −4.4% to −1.3%). Breast cancer remains the leading cause of death among young female patients, with CRC ranked second since 2023.

## Discussion

Similar to a recent US study,^1^ CRC has become the leading cause of cancer-related death among individuals younger than 50 years in Norway. However, this was not accompanied by increased mortality. Instead, the pattern appears to be attributed to decreased mortality of other major cancer types and the balance between increased incidence and survival of early-onset CRC. According to GLOBOCAN 2022 estimates, Norway has the third highest incidence of early-onset CRC worldwide, increasing by 66% from 1993 to 2022.^3^ In the same period, the 5-year relative survival increased by 19 percentage points among young patients.^4,5^ Failure to obtain a corresponding decrease in mortality might also be associated with more frequent diagnosis at advanced stage.^3^ Notably, decreased mortality was observed for young female patients with rectal cancer, and this coincided with improved treatment, including introduction of total mesorectal excision and increased use of preoperative chemoradiation. Norway has a publicly funded health care system and a national cancer registry providing unbiased, population-representative data. This might contribute to differences in observed mortality trends relative to studies from the United States, but a main limitation of this study is the relatively small size of the Norwegian population. Further investigation is needed to understand potential variations in mortality trends.

In Norway, only individuals older than 55 years are invited to participate in population-based screening (since 2022) or screening trials for CRC, and screening is unlikely to impact the mortality trends observed in this study.^7^ In the United States, the uptake of screening in individuals aged 45 to 49 years has increased after recent guideline changes.^8^ This was associated with increased incidence of local-stage CRC,^9^ although the potential impact on mortality remains to be seen.^10^ This reflects an increased awareness of the growing health burden of early-onset CRC. Improved understanding of disease etiology will be crucial to further adapting the prevention and management of CRC in young patients.

## Notes

Supplement.
Data Sharing Statement

## References

[1] Siegel RL, Wagle NS, Jemal A. Leading cancer deaths in people younger than 50 years. JAMA. 2026;335(7):632-634. doi:10.1001/jama.2025.25467 41569583 PMC12828652

[2] Siegel RL, Torre LA, Soerjomataram I, . Global patterns and trends in colorectal cancer incidence in young adults. Gut. 2019;68(12):2179-2185. doi:10.1136/gutjnl-2019-319511 31488504

[3] Ystgaard MF, Myklebust TÅ, Smeby J, . Early-onset colorectal cancer incidence in Norway: a national registry-based study (1993-2022) analyzing subsite and morphology trends. ESMO Gastrointest Oncol. 2024;7:100065. doi:10.1016/j.esmogo.2024.100065 41646485 PMC12836505

[4] Larønningen S, Arvidsson G, Bray F, . NORDCAN: comparable cancer statistics for Denmark, Finland, Iceland, Norway, Sweden, the Faroe Islands, and Greenland. Accessed April 1, 2026. https://nordcan.iarc.fr/

[5] Engholm G, Ferlay J, Christensen N, . NORDCAN—a Nordic tool for cancer information, planning, quality control and research. Acta Oncol. 2010;49(5):725-736. doi:10.3109/02841861003782017 20491528

[6] Norwegian Institute of Public Health. Cancer Registry of Norway Statistics Bank. Accessed April 6, 2026. https://sb.kreftregisteret.no/

[7] Kaminski MF, Kalager M, Løberg M, ; NordICC Study Group*. Long-term effects of colonoscopy screening on colorectal cancer incidence and mortality: a multicountry, population-based randomised controlled trial. Lancet. 2026;407(10541):1787-1795. doi:10.1016/S0140-6736(26)00508-8 42102826

[8] Harris AH, Murphy HR, McDowell M, Wright ME, Hughes MC. Facility-based uptake of colorectal cancer screening in 45- to 49-year-olds after US guideline changes. JAMA Netw Open. 2025;8(11):e2541330. doi:10.1001/jamanetworkopen.2025.41330 41186951 PMC12587196

[9] Schafer EJ, Sung H, Star J, Bandi P, Smith RA, Siegel RL. Colorectal cancer incidence in US adults after recommendations for earlier screening. JAMA. 2025;334(9):824-826. doi:10.1001/jama.2025.9147 40758342 PMC12322822

[10] Chiu HM, Chen SLS, Su CW, . Long-term effectiveness associated with fecal immunochemical testing for early-age screening. JAMA Oncol. 2025;11(8):846-854. doi:10.1001/jamaoncol.2025.1433 40504543 PMC12163714

