# Supplementary material for: Temporal Trends in Cancer Mortality Among People Younger Than 50 Years in Norway
Source: JAMA Netw Open. 2026 Aug 14;9(8):e2629437. doi: 10.1001/jamanetworkopen.2026.29437 (PMC13476840; doi:10.1001/jamanetworkopen.2026.29437)
Supplement: Supplementary file 1 [file jamanetwopen-e2629437-s001.pdf]

Data Sharing Statement

Nunes. Temporal Trends in Cancer Mortality Among People Younger Than 50 Years in Norway. *JAMA Netw Open*. Published August 14, 2026.
doi:10.1001/jamanetworkopen.2026.29437

Data
Data available: No

Additional Information
Explanation for why data not available: This study used publicly available data as described in the Methods; no additional data is available.
